# Nuclei on the move: LINC complex-dependent and -independent mechanisms of nuclear migration in development

**DOI:** 10.1080/19491034.2026.2679812

**Published:** 2026-05-31

**Authors:** Selin Gümüşderelioğlu, G. W. Gant Luxton, Daniel A. Starr

**Affiliations:** Department of Molecular and Cellular Biology, University of California, Davis, Davis, CA, USA

**Keywords:** LINC, nuclear migration, nuclear envelope, nuclear-cytoskeleton interactions, nuclear mechanobiology

## Abstract

Nuclear positioning drives developmental processes from fertilization to neurogenesis. The linker of nucleoskeleton and cytoskeleton (LINC) complex connects nuclei to cytoskeletal force generators that power nuclear migration. Mutations in LINC complex components cause muscular dystrophies and neurodegenerative disorders, yet mechanisms controlling nuclear movement remain poorly understood. We summarize advances from three *C. elegans* developmental models in different mechanical environments: pronuclear migration across the vast zygotic cytoplasm, hyp7 hypodermal precursor nuclear migration where nuclei span nearly the width of the cell, and P-cell nuclear migration through narrow constrictions. These systems reveal multiple regulatory strategies for nuclear movement. Tissue-specific expression of distinct SUN-KASH combinations determines which cytoskeletal elements engage nuclei. Alternative splicing generates KASH isoforms with opposing motor preferences, and P-cell nuclear migration through constrictions requires four parallel pathways. These findings establish conserved mechanistic principles of nuclear migration, with implications for development, immune cell function, and cancer metastasis.

## Introduction

The precise positioning of nuclei is essential for numerous developmental processes, including pronuclear migration during fertilization, and for cellular architecture establishment in muscle and neuronal development [1,2]. Nuclear movement requires coordination between cytoskeletal force generators and linker of nucleoskeleton and cytoskeleton (LINC) complexes, which span the nuclear envelope to physically couple nuclei with the cytoplasm. Defects in LINC complex components cause neuromuscular and neurodegenerative disorders including amyotrophic lateral sclerosis [3–5], ataxia [5,6], DYT1 dystonia [7], lissencephaly [8], and Emery-Dreifuss Muscular Dystrophy [9].

How LINC complexes are regulated to move nuclei to appropriate subcellular locations remains poorly understood, although emerging evidence is beginning to implicate a couple of regulatory mechanisms. For example, torsinA, a luminal ATPases-associated with various cellular activities protein, remodels SUN-KASH interactions in the perinuclear space to regulate LINC complex assembly and dynamics during nuclear movement [10], and the nuclear envelope-enriched protein disulfide isomerase TMX4 can break the intermolecular SUN-KASH disulfide bond to weaken LINC complex interactions [11]. Nevertheless, how these and other regulatory inputs are coordinated to direct nuclei to specific subcellular destinations at appropriate developmental times requires further investigation.

LINC complexes comprise Sad1/UNC-84 (SUN) proteins spanning the inner nuclear membrane and Klarsicht/ANC-1/SYNE homology (KASH) proteins in the outer nuclear membrane [12]. SUN proteins interact with lamins and other nucleoplasmic components [13,14], while KASH proteins engage proteins in the cytoplasm [15]. The divergent cytoplasmic domains of KASH proteins can connect the nucleus with all three components of the cytoskeleton [16,17], or Rho-family GTPase signaling machinery [18–20]. This molecular diversity enables LINC complexes to serve multiple functions ranging from force transmission to signaling, but how cells regulate which cytoskeletal elements are engaged during specific developmental events remains a central question.

Nuclear migrations occur in diverse mechanical environments, from the relatively unobstructed cytoplasm of a one-celled zygote to tissue contexts where nuclei must squeeze through openings smaller than their resting diameter. As the largest and stiffest organelle, the nucleus presents challenges when cells migrate through constrained spaces, as occurs during immune cell extravasation and cancer metastasis [21]. *In vitro* studies have revealed remarkable nuclear deformability and characterized cellular responses to nuclear envelope damage during severe deformation [22–25]. Interestingly, dendritic cells use their nucleus as a mechanical gauge to select paths of least resistance during interstitial migration, underscoring that nuclear mechanics actively dictate cellular migration strategies rather than merely constraining them [25]. However, better understanding of how LINC complexes transfer forces from the cytoskeleton to nuclei to coordinate nuclear movement across varied contexts requires genetically tractable *in vivo* models.

This review integrates recent findings to propose that cells regulate nuclear migration through a hierarchy of control mechanisms matched to mechanical challenges encountered during nuclear migration. In relatively unobstructed environments, tissue-specific expression of single LINC complex combinations suffices. As mechanical demand increases, requiring bidirectional motor coordination or movement through confined spaces, cells layer on additional regulatory mechanisms. These mechanisms include alternative splicing to modulate motor activity, and utilization of additional pathways that work with LINC complexes to ensure successful nuclear translocation. This framework suggests that therapeutic interventions targeting nuclear migration defects may need to address multiple mechanisms simultaneously.

## Nuclear migration faces various mechanical challenges throughout development

**Figure 1.**
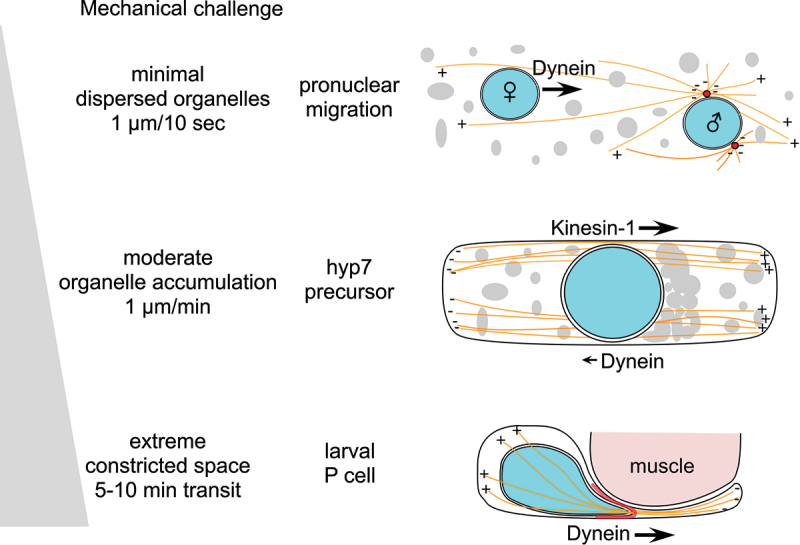
Three *C. elegans* models used to study nuclear migrations subject to varying mechanical challenges. (top)Female pronuclear migration in the newly fertilized zygote where the pronucleus moves through a vast cytoplasm with smaller, dispersed organelles. This is an environment with minimal mechanical challenge that allows nuclei to move at the speed of ~1 µm/10 sec. Dynein localizes to the cytosolic face of the pronucleus via a LINC complex comprising the SUN protein SUN-1 and the KASH protein ZYG-12, and captures microtubules (shown in orange) from the male pronuclear asters (centrosomes shown in red) to move the female pronucleus toward the male pronucleus. (middle) Schematic illustrating nuclear migration in an embryonic hyp7 precursor. Nuclei span nearly the width of the cell and midway through are challenged by an accumulation of organelles in front of the migrating nucleus (shown in gray). This creates a moderately challenging mechanical environment where nuclei move ~1 µm/minute. A LINC complex comprising the SUN protein UNC-84 and the KASH protein UNC-83c recruits both kinesin-1 and dynein to the nuclear envelope. Kinesin-1 is the main force driver moving the nuclei towards the plus-ends of microtubules (shown in orange, + ends to the right) while dynein aids nuclei to make a short backwards movement which allows the release of the accumulation so the nucleus can move past the organelles. (bottom) Schematic illustrating P-cell nuclear migration through narrow constrictions. The constricted passageway between body-wall muscles (shown in pink) and the cuticle (not shown) is only about 5% the width of the P-cell nucleus before migration, creating an extremely challenging mechanical environment for the nuclear movement. Four parallel pathways comprised of 1) UNC-84, UNC-83a/b, dynein; 2) CDC-42 actin networks (in pink at the constriction); 3) FLN-2 nuclear envelope reinforcement; and 4) CEC-4 peripheral heterochromatin work together to move the nuclei through the constriction and toward the ventral cord.

Comparison across multiple developmental contexts reveals a striking pattern: the number of parallel mechanisms required for successful nuclear migration correlates with the severity of mechanical challenges confronting nuclear migration (Figure 1). *C. elegans* offers an excellent system for studying nuclear migration because its optical transparency, powerful genetics, and well-characterized development, enable real-time observation and genetic dissection of nuclear movement through different mechanical environments *in vivo* [26,27]. Pronuclear migration through the relatively unobstructed cytoplasm in the large zygote primarily requires LINC complex-mediated dynein activity [28,29], while the extreme deformations of P-cell nuclear migration, where nuclei traverse an opening only 5% of their resting diameter (2–3 μm diameter of resting P-cell nuclei, with a constriction that may be as small as 100–200 nm) [30], demand four parallel pathways acting in concert. We propose that this multi-pathway coordination reflects a general principle where cells scale their mechanistic investment to match the mechanical challenge of nuclear translocation (Figure 1). This framework may explain why mammalian cells undergoing confined migration similarly activate multiple mechanisms including ESCRT-III-mediated nuclear envelope repair [22,24], perinuclear actin polymerization [23], and heterochromatin reorganization [31,32]. While nuclear envelope rupture during interphase is well-documented in cancer cells [24], its relevance during normal development remains less established and should be considered a potential, rather than definitive strategy, for mechanically challenged nuclear transit in non-transformed cells. Beyond a crowded or constrained cytoplasm, other factors such as cell type-specific chromatin organization, particularly changes in peripheral heterochromatin during development, and cytoskeletal architecture may also contribute to the observed variations in nuclear migration mechanisms, and these possibilities are briefly considered below.

There are at least five nuclear migration events dependent on LINC complexes during *C. elegans* development. Nuclei are localized to polarized positions in both the embryonic intestinal primordium [33,34] and in the migrating distal tip cell of the somatic gonad [35]. However, the relative mechanical challenges of nuclear positioning in these two cell types are not as clear. Here, we compare nuclear migration mechanisms across three developmental contexts (Figure 1) and discuss how the complexity of the migration machinery scales with the mechanical demands of each context.

### Context #1, pronuclear migration

Pronuclear migration brings the male and female genomes together before the first mitotic division, and defects cause severe chromosomal segregation errors [28,29]. In most animals, pronuclear migration is primarily driven by dynein motors [36] recruited to the nuclear envelope by KASH proteins [37]. In *C. elegans*, dynein generates forces at multiple locations. It maintains centrosome attachment to the male pronucleus [38], acts at the embryo cortex to center the asters [39], and localizes to female pronuclear surface where it captures microtubules from the male pronuclear asters to move the female pronucleus toward the male pronucleus [29] (Figure 1 top). A LINC complex comprising the SUN protein SUN-1 and the KASH protein ZYG-12 mediates both centrosome attachment and female pronuclear movement [27]. The female pronucleus moves at a steady and relatively quick pace of 1 µm/10 sec, suggesting a fairly unconstrained cytoplasm with dispersed organelles that pronuclei can easily migrate through.

### Context #2, Hyp7 precursor nuclear migration

During mid *C. elegans* embryogenesis, nuclei in the dorsal epithelial cells called hyp7 precursors migrate across the dorsal midline before these cells fuse to form the hyp7 syncytium [40]. This nuclear migration, at about 1 µm/min [41], is considerably slower than pronuclear migration, suggesting a more challenging environment to move through. Mutations in the LINC complex components *unc-83* (KASH) or *unc-84* (SUN) block this migration, leaving mislocalized nuclei in the dorsal cord [42]. In contrast to pronuclear migration, hyp7 nuclear movement depends primarily on kinesin-1, a heterotetramer of two UNC-116 heavy chains and two KLC-2 light chains [43]. A fusion protein directly connecting the UNC-83 transmembrane and KASH domains to KLC-2 rescues nuclear migration in *unc-83* null mutants [43], demonstrating that kinesin-1 provides the predominant driving force. Hyp7 nuclei, which nearly span the width of the cell, move in a bi-directional manner and often stall midway through migration when vesicles and lipid droplets accumulate in their path (Figure 1 middle); dynein then generates brief backward movements to clear the obstruction before kinesin-1 resumes forward transport [41]. UNC-83 contains a W-acidic motif (EWD) found in canonical kinesin-1 cargo adaptors, which activate kinesin-1 by binding kinesin light chain and releasing motor autoinhibition [17,44,45]. Mutating the EWD motif in UNC-83 causes severe hyp7 nuclear migration defects [45]. The mammalian KASH protein nesprin-4 contains a similar W-acidic (LEWD) motif required for kinesin-1 activation *in vitro* [46] and nuclear migration in ear hair cells [45], suggesting this is a conserved mechanism for KASH protein-mediated kinesin-1 recruitment and regulation.

### Context #3, larval P-cell nuclear migration

At hatching, L1 larvae have six epithelial blast cells called P cells on each lateral side of the animal. During mid L1, starting with the most anterior pair, P-cell nuclei migrate from lateral positions and intercalate to form a ventral row of 12 nuclei [30,47]. Following migration, P cells divide to generate vulval precursors, hypodermal cells, or neuronal lineages [47,48]. When P-cell nuclear migration fails, as observed in *unc-83* or *unc-84* mutants, animals exhibit egg-laying defects from missing vulval lineages and uncoordinated movement from reduced GABA neuron numbers [49]. This nuclear migration is the most difficult, as P-cell nuclei squeeze through a cellular constriction about 5% of their resting width. The kinetics of P-cell nuclear migration are difficult to determine, but it takes 5–10 minutes for P-cell nuclei to transit the narrow constriction [30].

In all three migration events, the LINC complex needs to interact with the nucleoskeleton. The nucleoplasmic N-terminal domain of the SUN protein UNC-84 directly binds *C. elegans* LMN-1, the sole *C. elegans* lamin [14]. Knockdown of *lmn-1* severely disrupts nuclear migration in zygotes, hyp7 precursors, and P cells, demonstrating the essential role of nucleo-cytoskeletal coupling [14,28,30,50,51]. Separation-of-function mutations reveal the specificity of this interaction: *unc-84(P91S)* specifically disrupts UNC-84 to LMN-1 binding, causing intermediate defects where some nuclei migrate normally, while others fail to initiate, and others stall mid-migration [14]. Conversely, *lmn-1(R204W)* severely disrupts nuclear migration while preserving other lamin functions in viability and motility; this allele specifically prevents UNC-84 localization to the nuclear envelope [50]. Lamins similarly bind SUN proteins in mammalian cells [52], suggesting a conserved role in anchoring LINC complexes to the nucleoskeleton.

These three migration events thus present a gradient of mechanical challenge (Figure 1). Pronuclei move through the relatively unobstructed cytoplasm of a one-celled embryo, hyp7 precursor nuclei that span nearly the width of the cell traverse cells with occasional cytoplasmic obstructions, and P-cell nuclei squeeze through constrictions approximately 5% of their diameter. Here, we focus on the mechanisms needed to navigate the gradient of mechanical challenges and address a fundamental question: do cells match the complexity of their nuclear migration machinery to the mechanical demand present in their environment? The sections that follow examine the regulatory mechanisms used in each context.

## Tissue-specific expression of LINC complex components

One mechanism for regulating which motor moves nuclei at different developmental stages is tissue-specific expression of LINC complex components. *C. elegans* has two SUN proteins: the canonical UNC-84, predominantly expressed in somatic cells after the 26-cell stage [53], and the divergent SUN-1, predominantly expressed in the germline and early embryo [29,54,55]. These SUN proteins partner with distinct KASH proteins. SUN-1 pairs with ZYG-12 in the germline and early embryo [37], while UNC-84 interacts with ANC-1, KDP-1, and UNC-83 [56–58].

Both ZYG-12 and UNC-83 recruit dynein to move nuclei toward microtubule minus ends, but at different developmental stages. ZYG-12 localizes to the female pronuclear envelope to drive migration toward the male pronucleus in the fertilized embryo [29], while UNC-83 recruits dynein to migrating P-cell nuclei in larvae [30,59]. ZYG-12 also functions specifically in the germline to tether dynein to meiotic prophase chromosomes for homolog pairing [54]. This regulatory theme is conserved in mammalian KASH proteins as nesprin-4 is narrowly expressed in select tissues [60,61], KASH5 is specific to meiotic prophase [62], JAW1/LRMP is enriched in lymphoid and taste receptor cells [63,64], and nesprins-1, −2, and −3 are more broadly expressed.

Beyond expression patterns, ZYG-12 and UNC-83 differ in how they engage dynein. UNC-83 functions as a classical dynein cargo adaptor, recruiting the motor through accessory proteins including BICD, NudEL, and Lis1 orthologs [17,27,65]. In contrast, ZYG-12 is itself a hook protein in the BICD family of dynein adaptors, enabling direct dynein engagement without additional accessory factors [29,66]. Interestingly, mammalian KASH5 recruits dynein more like UNC-83, as a classical cargo adaptor, despite serving a ZYG-12-like function in moving meiotic chromosomes [67].

Tissue-specific expression of LINC complex components provides the first regulatory layer, determining which cytoskeletal elements engage the nucleus in different developmental contexts. SUN-1/ZYG-12 LINC complexes recruit dynein in the early embryo; UNC-84/UNC-83 can recruit either dynein or kinesin-1 in later development. Nevertheless, expression patterns alone cannot explain how a single KASH protein, for example UNC-83, drives nuclei in opposite directions in different tissues. This requires a second regulatory layer, alternative splicing.

## Alternative ZYG-12 isoforms recruit dynein to pronuclei or centrosomes

**Figure 2.**
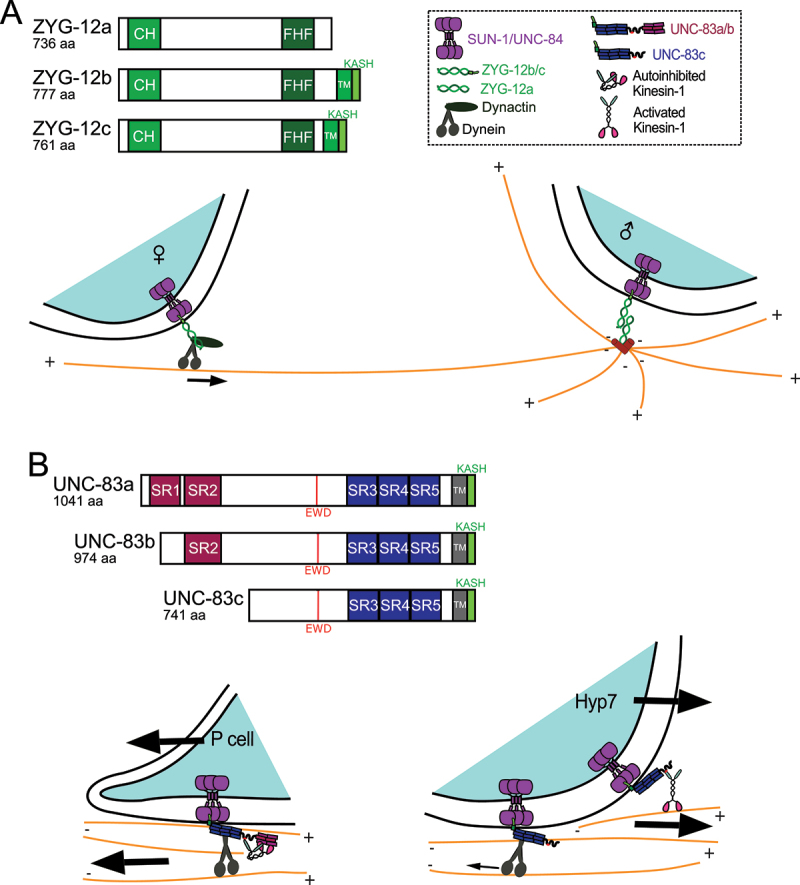
Tissue-specific expression of LINC complex components and alternative splicing of KASH proteins regulate which motor moves nuclei at different developmental stages. (A) There are three ZYG-12 isoforms. All isoforms contain calponin homology (CH) and FTS-Hook-FHIP (FHF) complex binding region while only ZYG-12b and ZYG-12c contain C-terminal transmembrane (TM) and KASH domains. The illustration shows the molecular mechanism used in pronuclear migration. The SUN protein SUN-1 (purple) and the KASH protein ZYG-12b/c (green) form a LINC complex spanning the nuclear envelopes of both female (on the left) and male (on the right) pronuclei. ZYG-12b/c binds to dynein and dynactin (both shown in dark gray). ZYG-12b/c binds to ZYG-12a on the male pronucleus. ZYG-12a is a component of centrosomes and the ZYG-12b/c to ZYG-12a interaction is necessary to connect centrioles (shown in red) to the male pronucleus. The female pronucleus uses dynein to move toward the male pronucleus along microtubules nucleated at the male pronucleus (shown in orange). (B) There are three isoforms of UNC-83. All three contain a transmembrane domain (TM) (gray), the KASH peptide (green), an EWD motif that interacts with KLC-2 (red line), and three spectrin-like repeats at the C-terminal (SR3, SR4 and SR5 shown in dark blue). Two additional spectrin repeats (SR1 and SR2 in magenta) are unique to UNC-83a/b. The illustrations show LINC complexes at the nuclear envelope. On the lower left, dynein drives migration of larval P-cell nuclei (cyan) toward the minus-ends of microtubules (orange, minus on left). The SUN protein UNC-84 (shown in purple) and the KASH protein UNC-83a/b (with all five spectrin repeats shown in dark blue and magenta) form a LINC complex on the nuclear envelope. UNC-83a/b activates dynein (dark gray) and/or inhibits kinesin-1 (green and pink), making dynein the main driver of larval P-cell nuclear migration. On the right, the kinesin-1-mediated migration of embryonic hyp7 nuclei (cyan) toward the plus-ends of microtubules (shown in orange, plus ends to the right). The short isoform UNC-83c (with only three spectrin repeats shown in dark blue) activates kinesin-1 as the main motor driving embryonic hyp7 nuclear migration while also recruiting dynein to move the nucleus backwards to pass large organelle accumulations in the cytoplasm.

The KASH protein ZYG-12 is regulated through alternative isoforms that direct dynein to distinct subcellular locations during pronuclear migration. The *zyg-12* locus encodes three isoforms with distinct domain structures. ZYG-12a, ZYG-12b, and ZYG-12c comprise 736, 777, and 761 amino acids, respectively; ZYG-12b and ZYG-12c contain C-terminal transmembrane and KASH domains, while ZYG-12a lacks these domains entirely [29,66] (Figure 2(A)). During pronuclear migration, ZYG-12b/c isoforms localize to the outer nuclear membrane of both pronuclei through an interaction with SUN-1, forming LINC complexes that recruit dynein for nuclear movement. ZYG-12a instead localizes to centrosomes, where it binds ZYG-12b/c through homotypic coiled-coil interactions to maintain centrosome – pronuclear attachment [29]. This isoform-specific targeting enables a single locus to coordinate dynein activity at multiple sites during fertilization (Figure 2(A)).

KASH-less isoforms generated by alternative splicing represent an understudied aspect of KASH protein biology. Beyond centrosomal targeting, KASH-less ZYG-12 isoforms localize to early endosomes in *C. elegans* epithelia [66]. Similarly, KASH-less isoforms of *Drosophila* Klarsicht target lipid droplets [68,69]. The *C. elegans* giant KASH protein ANC-1 can maintain nuclear anchorage, ER morphology, and cytoplasmic biophysical properties without its KASH domain [70,71]. Mammalian giant KASH proteins nesprin-1 and −2 possess numerous tissue-specific isoforms [72–74], including KASH-less isoforms that lack the outer nuclear membrane-targeting KASH domain [72,75]. The KASH-independent functions of nesprin isoforms are not fully understood.

Alternative splicing of ZYG-12 enables spatial coordination of dynein activity during a single migration event. The *unc-83* locus employs a related but distinct strategy by using alternative isoforms to select between opposing motors in different tissues.

## Alternative UNC-83 isoforms engage different microtubule motors

Nuclei move in opposite directions along microtubules in hyp7 precursors versus larval P cells, despite both requiring UNC-83. Embryonic hyp7 precursor nuclei move toward microtubule plus ends in a kinesin-1-dependent manner [41,43], while larval P-cell nuclei migrate toward microtubule minus ends using dynein [30,65]. This directional switch is controlled by tissue-specific expression of *unc-83* isoforms [76]. At least three UNC-83 isoforms exist: UNC-83a, UNC-83b, and UNC-83c, comprising 1041, 974 and 741 amino acid residues, respectively (Figure 2(B)). All three share the same C-terminal 741 residues, including the KASH domain and a W-acidic kinesin-1-binding motif [33,76], but differ in their N-termini and are expressed from different promoters [33]. Consistent with its functional roles, UNC-83c is the predominant isoform expressed in embryonic hyp7 precursors [76]. *unc-83* null mutations eliminate all three isoforms and block both nuclear migration events. In contrast, mutations disrupting only the longer UNC-83a/b while preserving the UNC-83c short isoform permit normal hyp7 nuclear migration, but disrupt P-cell nuclear migration [33]. These genetic data support a model in which the short UNC-83c isoform activates kinesin-1 in hyp7 precursor, while the longer UNC-83a/b isoforms inhibit kinesin-1 in P cells, allowing dynein activity to predominate [76] (Figure 2(B)).

The short UNC-83c isoform activates kinesin-1 via its EWD W-acidic motif, which binds KLC-2 with high affinity to release motor autoinhibition [76]. The long UNC-83a/b isoforms contain an additional 301-residue N-terminal domain that inhibits kinesin-1 through two distinct mechanisms: it directly binds the kinesin heavy chain UNC-116 to block motor activation, and it reduces overall affinity of the isoform for KLC-2 by more than 10-fold relative to UNC-83c [76]. Thus, the additional N-terminal region in the long isoforms overrides kinesin-1 activation by the EWD motif, allowing dynein to predominate in P cells.

In contrast, UNC-83 functions predominantly through dynein during P-cell nuclear migration. Knockdown of the dynein adaptor proteins NudE (NUD-2) or Bicd1 (BICD-1) causes significant P-cell nuclear migration defects [30]. Furthermore, auxin-induced degradation of the dynein heavy chain (DHC-1) in P cells similarly disrupts nuclear migration [59], confirming that UNC-83 acts as a dynein adaptor protein. Precise deletions of the UNC-83a/b-specific N-termini block P-cell nuclear migration, suggesting that this region is necessary for dynein-mediated nuclear movement [76]. In addition, *in vitro* assays further revealed that the UNC-83a-specific N-terminal fragment inhibits kinesin-1 by directly binding to the kinesin heavy chain UNC-116^76^. Thus, in the presence of the long UNC-83a/b isoforms, kinesin-1 is inhibited, allowing dynein to serve as the primary force generator.

Mammalian giant KASH proteins nesprin-1 and −2 also bind to both kinesin-1 and dynein, suggesting mechanistic conservation [17,77]. Indeed, nesprin-2 has been shown to simultaneously engage kinesin-1 and dynein through structurally distinct, proximal binding sites to coordinate bidirectional nuclear movement during neuronal migration [78,79]. Recent reconstitution studies revealed that mammalian nesprin-2 not only activates kinesin-1 but also bundles F-actin and mediates microtubule-actin crosstalk [80], suggesting KASH proteins may coordinate multiple cytoskeletal systems during nuclear movement. How these KASH proteins regulate the activity balance between kinesin-1 and dynein motors requires further investigation. Notably, the UNC-83a/b N-terminal domain that inhibits kinesin-1 is predicted to contain two divergent spectrin-like repeats [76]. Perhaps some of the numerous spectrin-like repeats in nesprin-1 or −2, which are differentially included across tissue-specific isoforms [74] might play analogous roles in mammalian cells.

Alternative splicing adds a second regulatory layer to nuclear migration, enabling motor selection without changing LINC complex composition. Together, tissue-specific expression and isoform regulation provide precise control over nuclear movement in contexts where the mechanical challenge is moderate. But what happens when nuclei face severe deformation? Examining P-cell nuclear migration reveals that extreme mechanical challenges demand a different strategy, pathway redundancy.

## Multiple pathways work together to move nuclei through constricted spaces

**Figure 3.**
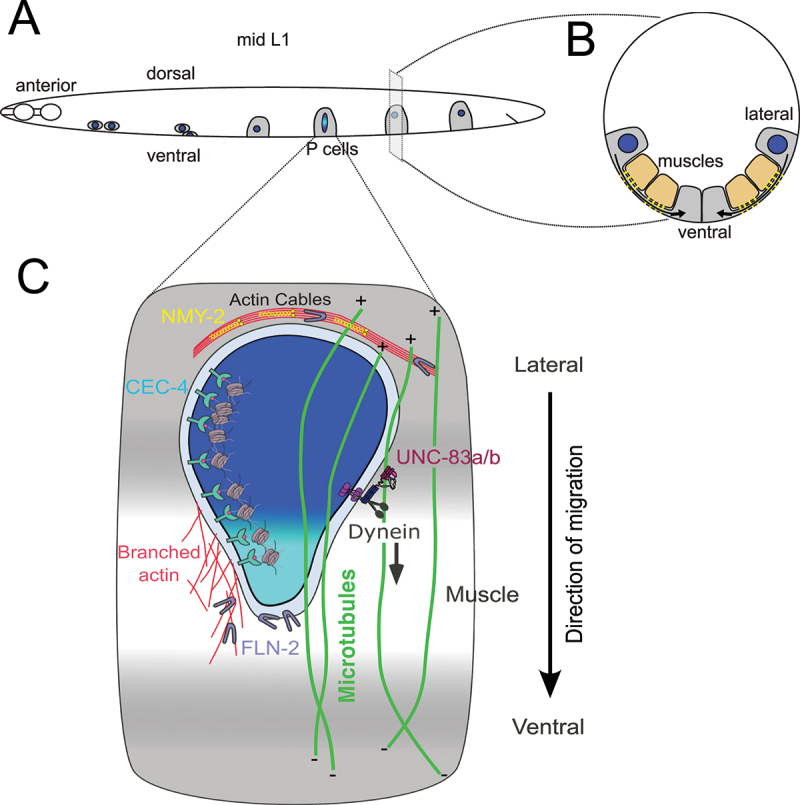
Multiple pathways contribute to P-cell nuclear migration through a constricted space. (A) A lateral view of the surface hypodermis in mid L1 larval stage. Six P-cell (gray) nuclei (blue) on each side of the animal migrate from their lateral position into the ventral cord. Anterior nuclei migrate first, so a pair of P-cell nuclei (one from each side) is shown on the anterior (left) of the animal, while more posterior cells represent earlier stages of nuclear migration (and their nuclei are represented as dark blue because they are not in the constricted space). The 4th P cell is in mid-migration and the light blue shading in the nucleus indicates the portion of the nucleus that has been flattened as it traverses the constricted space between body-wall muscles and the cuticle. The dark blue parts of the nucleus are outside of the constricted space. (B) A transverse cross section through a L1 P cell before nuclear migration. The nucleus (dark blue) has yet to move through the constriction between body-wall muscles and the cuticle. The constriction is about 5% (100–200 nm) of the width of the nucleus. The arrows indicate the paths of nuclear migration from lateral toward ventral. (C) Lateral surface of an L1 larval P cell in mid nuclear migration. The P-cell is gray, but the middle of the P cell is white to represent the constricted space (which is in the plane of the image). The body-wall muscle is immediately behind the lateral part of the P cell. P-cell nuclei traverse the narrow space between the muscle on the inside and the overlying cuticle on the outside of the P cell. Constricted migration is required even though the outer boundary of the P cell may not appear dramatically deformed. The muscle is not shown directly but is represented by the gray-to-white gradient behind the P cell, where the gradient reflects the muscle receding behind the plane of the constriction. Most of the nucleus is dark blue as it has yet to enter the constricted space. The part of the nucleus that has just entered the constricted space is shown in light blue. Multiple pathways work together to move P-cell nuclei. A LINC complex with UNC-83a/b favors dynein to move nuclei toward the minus ends of microtubules (green). Actin networks (red) and non-muscle myosin 2 (NMY-2; yellow) help the nucleus squeeze into the constriction, although the exact location of these networks is poorly described. FLN-2 (depicted as light purple boomerang shapes) may function at the nuclear envelope or elsewhere to protect the nucleus from rupture. Finally, CEC-4 (blue) anchors H3K9me3 (pink) heterochromatin (gray) at the periphery of the nucleus to maintain nuclear integrity. See text for details.

P-cell nuclear migration represents an extreme mechanical challenge in development and accordingly deploys a complex regulatory apparatus. During larval development, P-cell nuclei traverse an opening of only 100–200 nm between body wall muscles and the hypodermis, approximately 5% of nuclear width, to reach the ventral cord (Figure 3(A, B)) [30]. This challenge parallels confined cell migration in mammals [81]. In the case of P-cell nuclear migration, the LINC complex does not act alone. Many P-cell nuclei migrate normally in *unc-83* or *unc-84* null mutants [33,82], suggesting that additional mechanisms contribute to this process. Recent studies discussed below have identified three distinct pathways that function parallel to LINC complexes, revealing that cells meet extreme mechanical challenge with pathway redundancy rather than simply stronger versions of the same machinery.

A forward genetic screen identified pathways parallel to LINC complexes by exploiting the temperature-dependent P-cell nuclear migration defect of *unc-83* and *unc-84* null animals [83]. At the permissive 15°C, most P-cell nuclei migrate successfully, but at the restrictive 25°C, over half fail to migrate and the cells die [33,82]. Screening *unc-84* null animals at 15°C for enhancers that increased migration failure revealed genes with partially redundant functions missed in the original screens that identified *unc-83* and *unc-84* [83].

The enhancer screen identified CGEF-1, a guanine nucleotide exchange factor for the small rho GTPase CDC-42, and TOCA-1 (transducer of CDC-42 activity), which contains a predicted actin-nucleating domain and a membrane-binding F-BAR domain [83,84]. These findings implicated a branched-actin pathway regulated by CDC-42, functioning parallel to the LINC complex pathway. Auxin-induced degradation of essential proteins revealed additional components including CDC-42 itself, the ARP2/3 complex member ARX-3, and non-muscle myosin II (NMY-2) [84]. These data suggest a model in which CDC-42 activation drives localized branched-actin nucleation through TOCA-1 and ARP2/3, followed by NMY-2-mediated contractile forces. An intriguing model is that the branched actin network functions at the leading edge of the nucleus as it enters the constriction to facilitate nuclear deformation to enable entry into the narrow passageway. The involvement of the F-BAR domain protein TOCA-1 is interesting, as F-BAR proteins both sense and induce membrane curvature [85,86], suggesting this pathway may coordinate nuclear membrane remodeling during passage through constrictions. This model would be analogous to the proposed function of perinuclear actin polymerization described in migrating dendritic cells [23]. We further propose that NMY-2-mediated actomyosin contractility provides posterior compressive force to move nuclei forward using a mechanism similar to that proposed for migrating neurons [87]. However, the intracellular site of action for branched actin or actomyosin contraction in P cells is unknown. Thus, whether these mechanisms function to deform the nucleus, to push it forward, or both remains to be investigated (see also Figure 3(C)).

The enhancer screen also identified FLN-2, a divergent filamin that maintains nuclear envelope integrity during P-cell nuclear migration (dark gray in Figure 3(C)) [88]. FLN-2 functions in a third pathway, as *fln-2* mutations further enhance the P-cell nuclear migration defect of *unc-84; cgef-1* double mutants [88]. FLN-2 may protect nuclei from mechanical challenges during constricted migration. Nuclear deformation during passage through narrow openings often causes nuclear rupture in cultured mammalian dendritic or breast cancer cells [22,24]. FLN-2 appears to prevent nuclear envelope failure during extreme deformation events [88], potentially functioning as a cellular shock absorber that permits dramatic nuclear shape changes without compromising barrier function. The underlying mechanisms remain to be explored.

A fourth pathway includes peripheral heterochromatin organization and its role in nuclear mechanics during migration. This pathway centers on CEC-4 (bright blue in Figure 3(C)) [89] an inner nuclear membrane protein that tethers H3K9me3-marked heterochromatin to the nuclear periphery [90]. Mutations in *cec-4* enhance phenotypes caused by LINC complex or actin pathway mutations, and simultaneous disruption of all four pathways produces penetrant P-cell nuclear migration failure [89]. These findings indicate that heterochromatin organization actively contributes to nuclear mechanical properties during migration, rather than merely responding passively to shape changes. Peripheral heterochromatin may mechanically reinforce the nuclear envelope, maintain nuclear shape during deformation, or protect genetic material during migration. Heterochromatin similarly safeguards nuclear integrity during mechanical stress in multiple mammalian cancer cell lines [31,32,91,92] and untethering heterochromatin from the nuclear envelope increases nuclear deformability in fission yeast [93], suggesting a conserved mechanism.

Both FLN-2 and CEC-4 are thought to exert their effects by modulating nuclear mechanical properties, which could modulate nuclear and cell migration, rather than actively drive nuclear movement. Thus, one could argue these two pathways indirectly function in nuclear migration. However, genetic characterization found that the loss of either FLN-2 or CEC-4 further enhances nuclear migration failure in sensitized backgrounds, culminating in near-penetrant failure when all four pathways are simultaneously disrupted. This demonstrates that they make essential contributions to nuclear transit through constrictions that qualify them as parallel pathways to the LINC and branched actin mechanisms. The exact mechanisms of the FLN-2 and CEC-4 pathways await further characterization.

The identification of four parallel pathways reveals the complexity of nuclear migration through constricted spaces. Rather than relying on a single mechanism, cells employ multiple complementary pathways to ensure successful nuclear translocation. This suggests that therapeutic interventions targeting nuclear migration may need to address multiple mechanisms simultaneously.

## Future directions

Nuclear migration is an evolutionarily conserved process essential for tissue morphogenesis [1]. Each new discovery enriches our mechanistic understanding of this complex process involving multiple pathways at distinct developmental stages, yet significant questions remain. Addressing them will require both technological innovation and integrating multiple model systems.

A fundamental gap concerns the quantitative parameters of nuclear movement. How many LINC complexes, adaptors, and motor proteins are needed to move a single nucleus, and do these numbers vary across migration contexts? The recent discovery that the giant KASH protein ANC-1 influences cytoplasmic mechanical properties [71] opens a new dimension to this question; does LINC complex density also affect the biophysical environment through which nuclei move? Combining *in vivo* nanorheology approaches with quantitative imaging of LINC complex components could address this.

The mechanisms regulating motor protein choice, recruitment, and activation remain partially understood. While UNC-83 isoforms differentially regulate kinesin-1 activity, the structural basis for this regulation is unknown. We predict that the N-terminal spectrin-like repeats in UNC-83a/b physically occlude the KLC-2 binding site or recruit inhibitory factors. This hypothesis is testable through structural analysis and reconstitution of motor-KASH protein complexes *in vitro*. Whether analogous mechanisms operate in mammalian cells, as nesprin-1 and −2 contain numerous differentially spliced spectrin-like repeats, remains an open question with potential therapeutic relevance.

The four parallel pathways identified in P-cell nuclear migration raise questions about pathway integration. How do cells coordinate LINC complex-dependent motor forces, actin polymerization, nuclear envelope reinforcement, and heterochromatin organization in space and time? Do these pathways communicate through shared regulators, or do they function independently with purely additive contributions? Optogenetic tools enabling acute pathway disruption during live imaging could distinguish between these models. Understanding pathway crosstalk may reveal vulnerability points for disease intervention.

These mechanisms have clear relevance to human pathology. Mutations in LINC complex components cause muscular dystrophies and neurodegeneration, while cancer cells must deform their nuclei to metastasize. The mechanical changes in immune cells observed during aging, including T-cell stiffening and reduced interstitial migration through altered nuclear organization [94], may reflect deterioration of the same pathways characterized here. Translating insights from *C. elegans* to mammalian disease models, and ultimately to therapeutic development, remains an important goal. The genetic tractability and optical transparency that have made *C. elegans* powerful for mechanistic discovery position it well for continued contributions to understanding nuclear migration in health and disease, particularly as we develop tools to measure and manipulate the mechanical properties of living cells.

## Data Availability

Data sharing is not applicable to this article as no new data were created or analyzed in this study.
